# The complete mitochondrial genome of *Metidiocerus* sp. (Hemiptera: Cicadellidae: Idiocerinae)

**DOI:** 10.1080/23802359.2020.1788438

**Published:** 2020-07-11

**Authors:** Xiao-Chen Di, Hai-Rong Dong, Liang-Chen-Yu Shan, Li-Li Tian, Bin Zhang

**Affiliations:** a College of Life Sciences and Technology, Inner Mongolia Normal University, Hohhot, P. R. China; b Department of Clinical Laboratory, First Hospital of Hohhot, Hohhot, P. R. China

**Keywords:** Cicadellidae, mitogenome, phylogeny, *Metidiocerus* sp.

## Abstract

The species *Metidiocerus* sp. belonging to the subfamily Idiocerinae (Hemiptera, *Cicadellidae*). Here, we sequenced and annotated the mitochondrial genome (mitogenome) of *Metidiocerus* sp. This mitogenome was 15,079 bp long and encoded 13 protein-coding genes (PCGs), 22 transfer *RNA* genes (tRNAs), and 2 ribosomal *RNA* unit genes (rRNAs), and one non-coding region. The nucleotide composition biases toward A and T, which together made up 77.4% of the entirety. All 13 PCGs were initiated by the ATN (ATG, ATT, ATA, and ATC) codon. All PCGs terminate with the stop codons TAA except for COX2, ND4, and ND1 ended with single T. A phylogenetic tree generated by the Bayesian method showed that *Metidiocerus* sp. is closely related to *Idiocerus salicis* and *Idiocerus herrichii* which enriched the mitochondrial genome data of Idiocerinae.

Idiocerinae is one of the largest groups of arboreal leafhoppers, comprising 105 genera and approximately 800 described species (Zhang and Webb 2019). The genus *Metidiocerus* was originally described by Ossiannilsson (1981) containing six species and distributed only in the Palearctic region. Kwon (1985) considered *Metidiocerus* as a subgenus of *Idiocerus* and described one new species, *Idiocerus (Metidiocerus) nigrolineatus*. *Metidiocerus* was elevated to generic status (Isaev 1988). This genus is characterized morphologically by having pair of dark brown longitudinal marks on face, lateral margins of genae slightly concave or less straight at apex, forewing with three subapical cells, hind femur with 2 + 0 apical setal formula, apophysis of style bearing 1–2 setae distally, male dorsal basal abdominal apodemes shorter than ventral ones (Ossiannilsson 1981; Kwon 1985).

In this study, we sequenced and annotated the complete mitochondrial DNA of the *Metidiocerus* sp. (MT554451) for the first time. The specimen of *Metidiocerus* sp. was collected in Shawan County, Xinjiang Uygur Autonomous Region, China in July 2019. The samples and voucher specimens (No. IMNU2019070603) were stored in 100% ethanol in the field and then stored at −30 °C in Inner Mongolia Normal University, China. The entire body without abdomen was shipped to Tsingke (Beijing, China) for genomic extraction. The mitogenome sequence of *Metidiocerus* sp. was generated using Illumina HiSeq 2000 Sequencing System. De novo assembly of clean reads was performed using SPAdes version 3.11.0 (Bankevich et al. 2012). The base composition was analyzed by MEGA version 7 (Kumar et al. 2016). Genes were annotated with the MITOS (Bernt et al. 2013) web server.

The mitogenome of *Metidiocerus* sp. consists of a 15,079 bp circular DNA molecule, with 42.1% A, 35.3% T, 12.3% C, and 10.3% G, which has an A/T bias (77.4%, A + T content). It contains 13 protein-coding genes (PCGs), 22 transfer RNA genes (tRNAs), and 2 ribosomal RNA unit genes (rRNAs), and one non-coding region. Most genes are encoded on heavy strand except for four PCGs (ND4, ND4L, ND5, and ND1) and eight tRNA genes (tRNA-Gln, Cys, Tyr, Phe, His, Pro, Leu, and Val). All 13 PCGs were initiated by the ATN (ATG, ATT, ATA, and ATC) codon. All PCGs terminate with the stop codons TAA and TAG except for COX2, ND4, and ND1 ended with single T. The 12S rRNA and 16S rRNA genes in the *Metidiocerus* sp. mitogenome were 751 and 640 bp in size. The sequenced partial non-coding region was 739 bp in length, located after the 12S rRNA.

All 13 PCGs sequences were extracted from the mitochondrial DNA sequences of 18 related taxa of Cicadellidae. The concatenated PCGs using Bayesian inference (BI) method in MrBayes version 3.2.1 (Ronquist and Huelsenbeck 2003; Ronquist et al. 2012) under the GTR + G model. The phylogenetic tree (Figure 1) showed that *Metidiocerus* sp. is closely related to *Idiocerus salicis* and *Idiocerus herrichii* which enriched the mitochondrial genome data of Idiocerinae. The nearly complete mitogenome of *Metidiocerus* sp. could provide important information for the further studies of *Metidiocerus* phylogeny.

**Figure 1.**
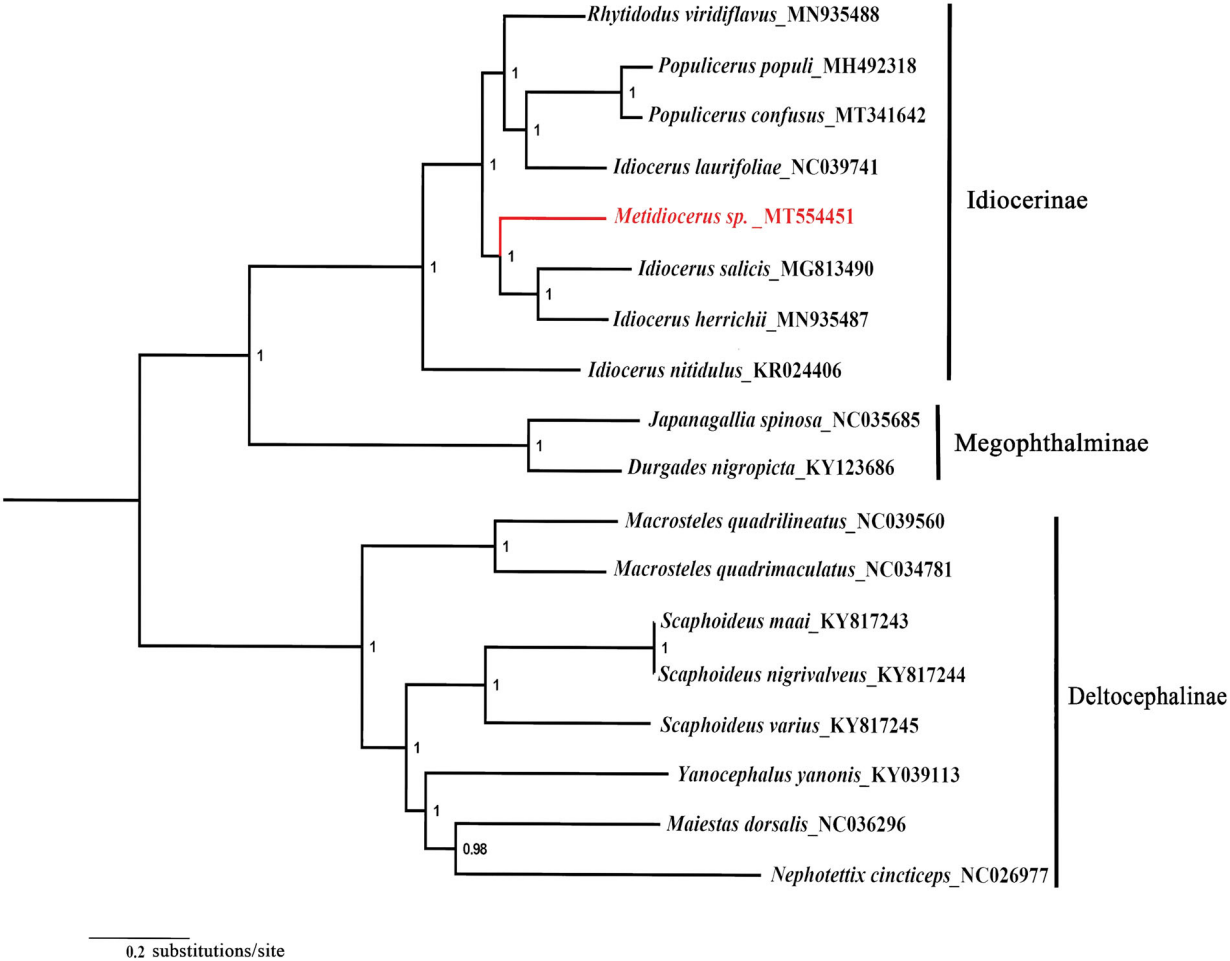
Phylogenetic tree (midpoint rooted) of the relationships among 18 species of Cicadellidae based on the nucleotide dataset of 13 PCGs. Numbers above the nodes indicate the posterior probabilities of Bayesian inference using MrBayes version 3.2.1 under the GTR + G model. Branch lengths represent means of the posterior distribution. The GenBank numbers of all species are shown in the figure.

## Data Availability

The authors confirm that the data supporting the finding of this study are available within its supplementary material. https://www.ncbi.nlm.nih.gov/nuccore/MT554451.
